# Crystal structure of 7-{[bis­(pyridin-2-ylmeth­yl)amino]­meth­yl}-5-chloro­quinolin-8-ol

**DOI:** 10.1107/S2056989015022410

**Published:** 2015-11-28

**Authors:** Koji Kubono, Kimiko Kado, Yukiyasu Kashiwagi, Keita Tani, Kunihiko Yokoi

**Affiliations:** a Division of Natural Sciences, Osaka Kyoiku University, Kashiwara, Osaka 582-8582, Japan; b Osaka Municipal Technical Research Institute, Osaka 536-8553, Japan

**Keywords:** crystal structure, 8-quinolinol, bis­(2-picol­yl)amine, hydrogen bonding, π–π inter­actions

## Abstract

In the title compound, C_22_H_19_ClN_4_O, the quinolinol moiety is almost planar [r.m.s. deviation = 0.012 Å]. There is an intra­molecular O—H⋯N hydrogen bond involving the hy­droxy group and a pyridine N atom forming an *S*(9) ring motif. The dihedral angles between the planes of the quinolinol moiety and the pyridine rings are 44.15 (9) and 36.85 (9)°. In the crystal, mol­ecules are linked *via* C—H⋯O hydrogen bonds forming inversion dimers with an *R*_4_^4^(10) ring motif. The dimers are linked by C—H⋯N hydrogen bonds, forming ribbons along [01-1]. The ribbons are linked by C—H⋯π and π–π inter­actions [inter-centroid distance = 3.7109 (11) Å], forming layers parallel to (01-1).

## Abstract 2

In the title compound, there is an intra­molecular O—H⋯N hydrogen bond forming an *S*(9) ring motif. In the crystal, mol­ecules are linked *via* C—H⋯O hydrogen bonds forming inversion dimers with an *R*_4_^4^(10) ring motif. The dimers are linked by C—H⋯N hydrogen bonds, forming ribbons along [01-1].

## Chemical context   

8-Quinolinol and its derivatives are well-known chelating reagents, forming fluorescent complexes with various metal ions, such as Al^3+^, Zn^2+^ and Cd^2+^ (Goon *et al.*, 1953 ▸; Valeur & Leray, 2000 ▸; Pohl & Anzenbacher, 2003 ▸). Bis(pyridin-2-ylmeth­yl)amine [di-(2-picol­yl)amine (DPA)] is an excellent ligand showing high selectivity for Zn^2+^, which plays important roles in biological, pathological and environmental processes (Berg & Shi, 1996 ▸; Bush *et al.*, 1994 ▸; Callender & Rice, 2000 ▸), and it is used to detect Zn^2+^ with low concentration in biological and environmental samples. Therefore, many fluorescence probes for Zn^2+^ bearing DPA as an ion-recognition site have been developed (Xue *et al.*, 2008 ▸; Chen *et al.*, 2011 ▸; Kwon *et al.*, 2012 ▸). We have synthesized a new fluorescence chemosensor, based on 8-quinolinol containing DPA *via* a two-step reaction, and herein we report on its synthesis and crystal structure.
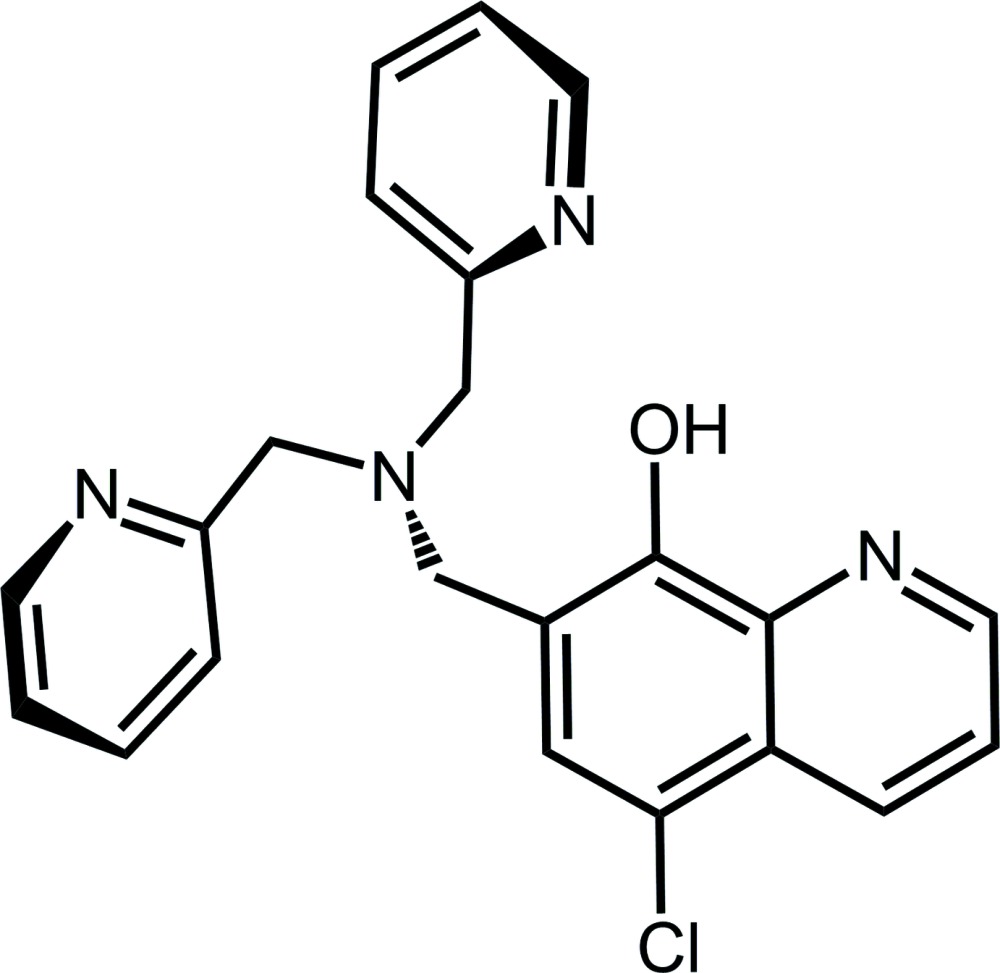


## Structural commentary   

**Figure 1:**
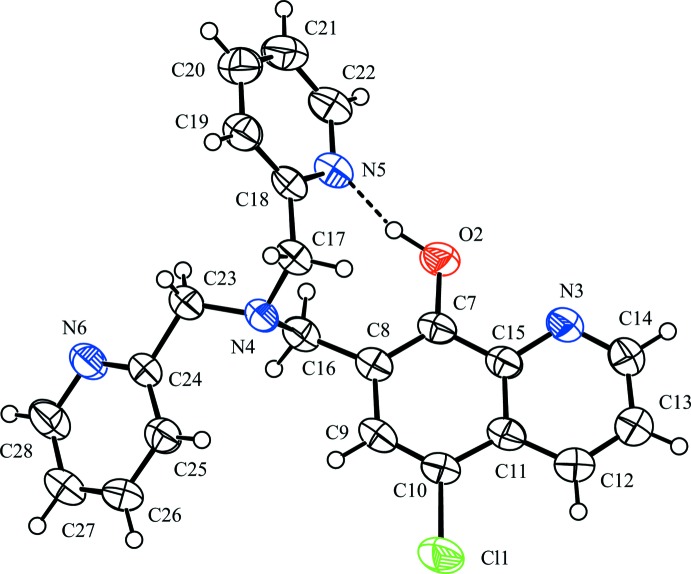
The mol­ecular structure of the title compound, showing the atom labelling. Displacement ellipsoids are drawn at the 50% probability level. The intra­molecular O—H⋯N hydrogen bond is shown as a dashed line (see Table 1 ▸).

The mol­ecular structure of the title compound, is shown in Fig. 1 ▸. There is an O—H⋯N intra­molecular hydrogen bond involving the hy­droxy group (O2—H2) and a pyridine N atom, N5, generating an *S*(9) ring motif (Fig. 1 ▸ and Table 1 ▸). The N(tertiaryamine)—C—C—N(pyridine) torsion angles, N4—C17—C18—N5 and N4—C23—C24—N6 are 75.0 (2) and 152.46 (19)°, respectively. The dihedral angle between the N5- and N6-containing pyridine rings pyridine rings is 80.97 (12)°, and they make dihedral angles of 44.15 (9) and 36.85 (9)°, respectively, with the quinolinol moiety.

## Supra­molecular features   

**Figure 2:**
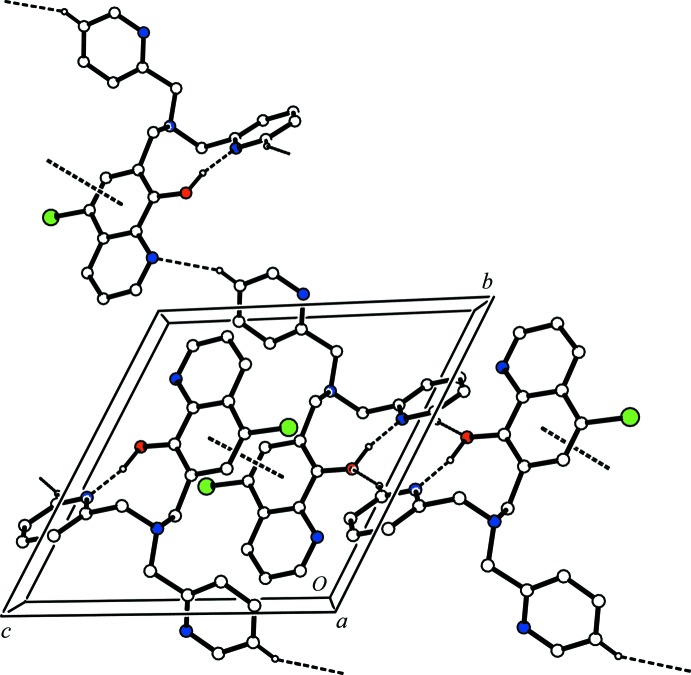
A view along the *a* axis of the crystal packing of the title compound. The hydrogen bonds (see Table 1 ▸) and π–π inter­actions are shown as dashed lines. H atoms not involved in these inter­actions have been omitted for clarity.

In the crystal, mol­ecules are linked *via* C—H⋯O hydrogen bonds, forming inversion dimers with an 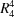
(10) ring motif (Fig. 2 ▸ and Table 1 ▸). The dimers are linked by C—H⋯N hydrogen bonds, forming ribbons along [01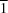]. The ribbons are linked by C—H⋯π (Table 1 ▸) and slipped parallel π–π inter­actions [*Cg*1⋯*Cg*1^i^, = 3.7109 (11) Å; *Cg*1 is the centroid of ring C7–C11/C15; inter-planar distance = 3.5518 (8) Å; slippage = 1.075 Å; symmetry code: (i) −*x*, −*y* + 1, −*z*], forming layers parallel to (01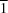) .

## Database survey   

A search of the Cambridge Structural Database (CSD, Version 5.36; Groom & Allen, 2014 ▸) for 8-quinolinols gave 387 hits, and for DPA, bis­(pyridine-2-ylmeth­yl)amine gave 4535 hits. A search for the fragment 2-[bis­(pyridin-2-ylmethyl-amino)-meth­yl]phenol gave 56 hits of which none contained 8-quinolinol. In the compounds that resemble the title compound, namely 2,6-bis­[bis­(pyridine-2-ylmeth­yl)amino­meth­yl]-4-tert-butyl­phenol (I) (Bjernemose & McKenzie, 2003 ▸), and 3-{[bis­(pyridin-2-ylmeth­yl)amino]­meth­yl}-2-hy­droxy-5-methyl­benzaldehyde (II) (Wang *et al.*, 2012 ▸), an intra­molecular bifurcated hydrogen bond is formed. The N—C—C—N torsion angles in the related compounds are −46.9 (2) and 152.7 (2)° in (I) and 48.35 (18) and −116.99 (15)° in (II), compared to 75.0 (2) and 152.46 (19)° in the title compound. The crystal structures of other compounds containing a fluorescent core and bis­(pyridine-2-ylmeth­yl)amine have been reported; for example one containing a fluorescein core (Wong *et al.*, 2009 ▸), and another a coumarin core (Kobayashi *et al.*, 2014 ▸).

## Synthesis and crystallization   

A suspension of paraformaldehyde (0.41 g, 14 mmol) and bis­(2-pyridyl­meth­yl)amine (1.99 g, 10 mmol) in 100 ml of MeOH was stirred for 18 h at room temperature. The solvent was removed under vacuum. To the product obtained was added 100 ml of toluene and 5-chloro-8-quinolinol (1.80 g, 10 mmol), and the mixture was heated for 24 h at 353 K. The solvent was removed under vacuum to give an oily product, which was crystallized from hexa­ne–di­chloro­methane. The crude solid was recrystallized from aceto­nitrile to obtain yellow crystals of the title compound (yield 55%; m.p. 380.4–382.6 K). HRMS (*m*/*z*): [*M* + 1]^+^ calculated, 391.1326; found, 391.1271. Analysis calculated for C_22_H_19_ClN_4_O: C 67.60, H 4.90, N 14.33%; found: C 67.50, H 5.01, N 14.37%.

## Refinement   

Crystal data, data collection and structure refinement details are summarized in Table 2 ▸. The hy­droxy H atom was located in a difference Fourier map and freely refined. The C-bound H atoms were positioned geometrically and refined using a riding model: C—H = 0.93–0.97 Å with *U*_iso_(H) = 1.2*U*_eq_(C).

## Supplementary Material

Crystal structure: contains datablock(s) global, I. DOI: 10.1107/S2056989015022410/su5241sup1.cif

Structure factors: contains datablock(s) I. DOI: 10.1107/S2056989015022410/su5241Isup2.hkl

Supporting information file. DOI: 10.1107/S2056989015022410/su5241Isup3.cml

CCDC reference: 1438483

Additional supporting information:  crystallographic information; 3D view; checkCIF report

## Figures and Tables

**Table 1:** Hydrogen-bond geometry (Å, °) *Cg*2 and *Cg*3 are the centroids of rings N5/C18–C22 and N6/C24–C28, respectively.

*D*—H⋯*A*	*D*—H	H⋯*A*	*D*⋯*A*	*D*—H⋯*A*
O2—H2⋯N5	1.04 (3)	1.66 (4)	2.689 (3)	168 (2)
C22—H22⋯O2^i^	0.93	2.46	3.348 (3)	160
C27—H27⋯N3^ii^	0.93	2.55	3.406 (3)	153
C17—H17b⋯*Cg*2^iii^	0.97	2.79	3.599 (3)	141
C23—H23*A*⋯*Cg*3^iv^	0.97	2.86	3.770 (3)	156

Symmetry codes: (i) 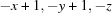; (ii) 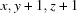; (iii) 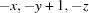; (iv) 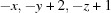.

**Table 2:** Experimental details

Crystal data	
Chemical formula	C_22_H_19_ClN_4_O
*M*_r_	390.86
Crystal system, space group	Triclinic, *P* 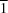
Temperature (K)	296
*a*, *b*, *c* (Å)	8.3170 (5), 11.5993 (7), 11.6135 (6)
α, β, γ (°)	116.8473 (13), 105.2809 (13), 92.0110 (17)
*V* (Å^3^)	948.68 (10)
*Z*	2
Radiation type	Mo *K*α
μ (mm^−1^)	0.22
Crystal size (mm)	0.30 × 0.20 × 0.10

Data collection	
Diffractometer	Rigaku R-AXIS RAPID
Absorption correction	Multi-scan (*ABSCOR*; Higashi, 1995 ▸)
*T*_min_, *T*_max_	0.769, 0.978
No. of measured, independent and observed [*F* ^2^ > 2.0σ(*F* ^2^)] reflections	9412, 4293, 2329
*R*_int_	0.023
(sin θ/λ)_max_ (Å^−1^)	0.648

Refinement	
*R*[*F* ^2^ > 2σ(*F* ^2^)], *wR*(*F* ^2^), *S*	0.036, 0.123, 1.09
No. of reflections	4293
No. of parameters	257
H-atom treatment	H atoms treated by a mixture of independent and constrained refinement
Δρ_max_, Δρ_min_ (e Å^−3^)	0.26, −0.24

Computer programs: *RAPID-AUTO* (Rigaku, 2006 ▸), *SIR92* (Altomare *et al.*, 1993 ▸), *SHELXL97* (Sheldrick, 2008 ▸), *PLATON* (Spek, 2009 ▸) and *CrystalStructure* (Rigaku, 2014 ▸).

## supplementary crystallographic information

### Crystal data

**Table:**

C_22_H_19_ClN_4_O	*Z* = 2
*M**_r_* = 390.86	*F*(000) = 408.00
Triclinic, *P*1	*D*_x_ = 1.368 Mg m^−^^3^
*a* = 8.3170 (5) Å	Mo *K*α radiation, λ = 0.71075 Å
*b* = 11.5993 (7) Å	Cell parameters from 5840 reflections
*c* = 11.6135 (6) Å	θ = 3.1–27.4°
α = 116.8473 (13)°	µ = 0.22 mm^−^^1^
β = 105.2809 (13)°	*T* = 296 K
γ = 92.0110 (17)°	Block, yellow
*V* = 948.68 (10) Å^3^	0.30 × 0.20 × 0.10 mm

### Data collection

**Table:**

Rigaku R-AXIS RAPID diffractometer	2329 reflections with *F*^2^ > 2.0σ(*F*^2^)
Detector resolution: 10.000 pixels mm^-1^	*R*_int_ = 0.023
ω scans	θ_max_ = 27.4°, θ_min_ = 3.1°
Absorption correction: multi-scan (*ABSCOR*; Higashi, 1995)	*h* = −10→10
*T*_min_ = 0.769, *T*_max_ = 0.978	*k* = −15→15
9412 measured reflections	*l* = −14→15
4293 independent reflections	

### Refinement

**Table:**

Refinement on *F*^2^	Secondary atom site location: difference Fourier map
*R*[*F*^2^ > 2σ(*F*^2^)] = 0.036	Hydrogen site location: inferred from neighbouring sites
*wR*(*F*^2^) = 0.123	H atoms treated by a mixture of independent and constrained refinement
*S* = 1.09	*w* = 1/[σ^2^(*F*_o_^2^) + (0.0488*P*)^2^ + 0.1777*P*] where *P* = (*F*_o_^2^ + 2*F*_c_^2^)/3
4293 reflections	(Δ/σ)_max_ < 0.001
257 parameters	Δρ_max_ = 0.26 e Å^−^^3^
0 restraints	Δρ_min_ = −0.24 e Å^−^^3^
Primary atom site location: structure-invariant direct methods	

### Special details

**Table:**

Geometry. Refinement was performed using all reflections. The weighted *R*-factor (*wR*) and goodness of fit (S) are based on F^2^. *R*-factor (gt) are based on F. The threshold expression of F^2^ > 2.0 sigma(F^2^) is used only for calculating *R*-factor (gt).
Refinement. Refinement was performed using all reflections. The weighted *R*-factor (*wR*) and goodness of fit (*S*) are based on *F*^2^. *R*-factor (gt) are based on *F*. The threshold expression of *F*^2^ > 2.0 σ(*F*^2^) is used only for calculating *R*-factor (gt).

### Fractional atomic coordinates and isotropic or equivalent isotropic displacement parameters (Å^2^)

**Table:**

	*x*	*y*	*z*	*U*_iso_*/*U*_eq_	
Cl1	0.20020 (9)	0.40251 (6)	0.58501 (6)	0.0724 (2)	
O2	0.3787 (2)	0.45209 (15)	0.14798 (14)	0.0563 (4)	
N3	0.3767 (2)	0.22221 (18)	0.14868 (17)	0.0560 (5)	
N4	0.1525 (2)	0.72637 (15)	0.33905 (15)	0.0432 (4)	
N5	0.2432 (2)	0.61932 (17)	0.06500 (16)	0.0500 (4)	
N6	0.2990 (2)	1.07071 (18)	0.58796 (17)	0.0562 (5)	
C7	0.3408 (2)	0.4468 (2)	0.25274 (18)	0.0444 (5)	
C8	0.3075 (2)	0.5531 (2)	0.35655 (19)	0.0440 (5)	
C9	0.2637 (3)	0.5344 (2)	0.45812 (19)	0.0488 (5)	
C10	0.2564 (3)	0.4177 (2)	0.45746 (19)	0.0474 (5)	
C11	0.2949 (2)	0.3074 (2)	0.35528 (18)	0.0451 (5)	
C12	0.2940 (3)	0.1827 (2)	0.3486 (2)	0.0546 (5)	
C13	0.3335 (3)	0.0841 (2)	0.2454 (2)	0.0642 (6)	
C14	0.3733 (3)	0.1088 (2)	0.1483 (2)	0.0645 (6)	
C15	0.3387 (2)	0.3236 (2)	0.25179 (18)	0.0439 (5)	
C16	0.3190 (3)	0.6889 (2)	0.3710 (2)	0.0469 (5)	
C17	0.0484 (3)	0.6472 (2)	0.19651 (18)	0.0453 (5)	
C18	0.1068 (3)	0.67040 (19)	0.09391 (18)	0.0440 (5)	
C19	0.0266 (3)	0.7400 (2)	0.0336 (2)	0.0542 (5)	
C20	0.0860 (3)	0.7593 (3)	−0.0582 (2)	0.0646 (6)	
C21	0.2264 (3)	0.7087 (3)	−0.0865 (2)	0.0637 (6)	
C22	0.2999 (3)	0.6399 (2)	−0.0238 (2)	0.0577 (6)	
C23	0.1681 (3)	0.8665 (2)	0.3842 (2)	0.0549 (6)	
C24	0.2147 (3)	0.9495 (2)	0.53687 (19)	0.0463 (5)	
C25	0.1679 (3)	0.9048 (2)	0.6174 (2)	0.0563 (6)	
C26	0.2149 (3)	0.9865 (2)	0.7564 (2)	0.0600 (6)	
C27	0.3044 (3)	1.1103 (2)	0.8101 (2)	0.0570 (6)	
C28	0.3404 (3)	1.1481 (2)	0.7224 (2)	0.0614 (6)	
H2	0.329 (3)	0.526 (3)	0.129 (3)	0.098 (9)*	
H9	0.23914	0.60515	0.52745	0.0585*	
H12	0.26668	0.16817	0.41415	0.0655*	
H13	0.33386	0.00152	0.23959	0.0771*	
H14	0.39931	0.0399	0.07819	0.0774*	
H16A	0.38532	0.7515	0.46354	0.0563*	
H16B	0.37804	0.69349	0.31109	0.0563*	
H17A	−0.06633	0.66426	0.18846	0.0544*	
H17B	0.04458	0.55522	0.17233	0.0544*	
H19	−0.06765	0.77418	0.05442	0.0651*	
H20	0.03192	0.80564	−0.10001	0.0776*	
H21	0.27022	0.72111	−0.14678	0.0764*	
H22	0.39441	0.60539	−0.04348	0.0692*	
H23A	0.06141	0.88388	0.34197	0.0659*	
H23B	0.25394	0.89247	0.35362	0.0659*	
H25	0.10548	0.82092	0.57873	0.0676*	
H26	0.18619	0.95777	0.81246	0.0720*	
H27	0.33951	1.16686	0.90321	0.0684*	
H28	0.39751	1.2333	0.75867	0.0737*	

### Atomic displacement parameters (Å^2^)

**Table:**

	*U*^11^	*U*^22^	*U*^33^	*U*^12^	*U*^13^	*U*^23^
Cl1	0.0990 (5)	0.0762 (4)	0.0553 (4)	0.0150 (4)	0.0451 (3)	0.0312 (3)
O2	0.0698 (10)	0.0665 (10)	0.0499 (8)	0.0272 (8)	0.0355 (8)	0.0321 (8)
N3	0.0734 (13)	0.0579 (12)	0.0446 (10)	0.0263 (10)	0.0282 (9)	0.0247 (9)
N4	0.0515 (10)	0.0396 (9)	0.0336 (8)	0.0077 (8)	0.0159 (7)	0.0123 (8)
N5	0.0525 (11)	0.0546 (11)	0.0389 (9)	0.0099 (9)	0.0180 (8)	0.0169 (9)
N6	0.0747 (13)	0.0454 (11)	0.0413 (10)	0.0005 (9)	0.0243 (9)	0.0122 (9)
C7	0.0422 (11)	0.0567 (13)	0.0350 (10)	0.0116 (10)	0.0153 (9)	0.0206 (10)
C8	0.0415 (11)	0.0493 (12)	0.0364 (10)	0.0063 (9)	0.0121 (8)	0.0167 (9)
C9	0.0504 (12)	0.0543 (13)	0.0336 (10)	0.0101 (10)	0.0165 (9)	0.0125 (10)
C10	0.0511 (12)	0.0551 (14)	0.0363 (10)	0.0075 (10)	0.0161 (9)	0.0208 (10)
C11	0.0416 (11)	0.0543 (13)	0.0347 (10)	0.0079 (10)	0.0108 (8)	0.0181 (10)
C12	0.0622 (14)	0.0598 (15)	0.0463 (12)	0.0117 (11)	0.0188 (11)	0.0281 (12)
C13	0.0846 (18)	0.0573 (15)	0.0593 (14)	0.0216 (13)	0.0287 (13)	0.0308 (13)
C14	0.0896 (18)	0.0579 (15)	0.0530 (13)	0.0316 (13)	0.0333 (13)	0.0250 (12)
C15	0.0437 (11)	0.0522 (13)	0.0352 (10)	0.0132 (10)	0.0143 (9)	0.0192 (10)
C16	0.0478 (12)	0.0478 (12)	0.0364 (10)	0.0022 (10)	0.0140 (9)	0.0129 (9)
C17	0.0450 (11)	0.0461 (12)	0.0361 (10)	0.0051 (9)	0.0135 (9)	0.0122 (9)
C18	0.0441 (11)	0.0431 (11)	0.0305 (9)	0.0027 (9)	0.0095 (8)	0.0075 (9)
C19	0.0544 (13)	0.0585 (14)	0.0441 (11)	0.0130 (11)	0.0146 (10)	0.0201 (11)
C20	0.0732 (17)	0.0701 (16)	0.0520 (13)	0.0108 (13)	0.0155 (12)	0.0326 (13)
C21	0.0717 (16)	0.0737 (17)	0.0466 (12)	0.0020 (13)	0.0214 (12)	0.0288 (13)
C22	0.0566 (14)	0.0681 (15)	0.0446 (12)	0.0090 (12)	0.0230 (10)	0.0203 (12)
C23	0.0801 (16)	0.0432 (13)	0.0376 (11)	0.0105 (11)	0.0217 (11)	0.0144 (10)
C24	0.0585 (13)	0.0413 (12)	0.0363 (10)	0.0111 (10)	0.0191 (9)	0.0137 (9)
C25	0.0781 (16)	0.0463 (13)	0.0465 (12)	0.0078 (11)	0.0280 (11)	0.0193 (11)
C26	0.0820 (17)	0.0648 (16)	0.0449 (12)	0.0187 (13)	0.0331 (12)	0.0283 (12)
C27	0.0613 (14)	0.0620 (15)	0.0355 (11)	0.0108 (12)	0.0189 (10)	0.0113 (11)
C28	0.0685 (16)	0.0535 (14)	0.0442 (12)	−0.0039 (12)	0.0215 (11)	0.0076 (11)

### Geometric parameters (Å, º)

**Table:**

Cl1—C10	1.743 (3)	C21—C22	1.366 (4)
O2—C7	1.361 (3)	C23—C24	1.514 (3)
N3—C14	1.313 (4)	C24—C25	1.382 (4)
N3—C15	1.368 (3)	C25—C26	1.384 (3)
N4—C16	1.470 (3)	C26—C27	1.369 (4)
N4—C17	1.466 (2)	C27—C28	1.370 (4)
N4—C23	1.454 (3)	O2—H2	1.04 (3)
N5—C18	1.349 (3)	C9—H9	0.930
N5—C22	1.347 (4)	C12—H12	0.930
N6—C24	1.334 (3)	C13—H13	0.930
N6—C28	1.338 (3)	C14—H14	0.930
C7—C8	1.381 (3)	C16—H16A	0.970
C7—C15	1.424 (4)	C16—H16B	0.970
C8—C9	1.422 (4)	C17—H17A	0.970
C8—C16	1.504 (3)	C17—H17B	0.970
C9—C10	1.349 (4)	C19—H19	0.930
C10—C11	1.416 (3)	C20—H20	0.930
C11—C12	1.412 (4)	C21—H21	0.930
C11—C15	1.429 (4)	C22—H22	0.930
C12—C13	1.359 (3)	C23—H23A	0.970
C13—C14	1.394 (5)	C23—H23B	0.970
C17—C18	1.521 (4)	C25—H25	0.930
C18—C19	1.375 (4)	C26—H26	0.930
C19—C20	1.384 (4)	C27—H27	0.930
C20—C21	1.376 (4)	C28—H28	0.930
			
C14—N3—C15	117.7 (2)	N6—C28—C27	124.4 (2)
C16—N4—C17	113.69 (15)	C7—O2—H2	112.4 (18)
C16—N4—C23	111.51 (16)	C8—C9—H9	118.825
C17—N4—C23	112.33 (18)	C10—C9—H9	118.839
C18—N5—C22	117.9 (2)	C11—C12—H12	120.293
C24—N6—C28	117.2 (2)	C13—C12—H12	120.294
O2—C7—C8	123.5 (2)	C12—C13—H13	120.515
O2—C7—C15	116.17 (17)	C14—C13—H13	120.514
C8—C7—C15	120.4 (2)	N3—C14—H14	117.649
C7—C8—C9	118.4 (2)	C13—C14—H14	117.655
C7—C8—C16	124.0 (2)	N4—C16—H16A	108.956
C9—C8—C16	117.66 (18)	N4—C16—H16B	108.959
C8—C9—C10	122.34 (19)	C8—C16—H16A	108.959
Cl1—C10—C9	119.55 (16)	C8—C16—H16B	108.962
Cl1—C10—C11	119.4 (2)	H16A—C16—H16B	107.759
C9—C10—C11	121.1 (2)	N4—C17—H17A	108.296
C10—C11—C12	124.8 (2)	N4—C17—H17B	108.295
C10—C11—C15	117.6 (2)	C18—C17—H17A	108.301
C12—C11—C15	117.59 (18)	C18—C17—H17B	108.301
C11—C12—C13	119.4 (3)	H17A—C17—H17B	107.402
C12—C13—C14	119.0 (3)	C18—C19—H19	119.991
N3—C14—C13	124.7 (2)	C20—C19—H19	119.983
N3—C15—C7	118.2 (2)	C19—C20—H20	120.561
N3—C15—C11	121.6 (2)	C21—C20—H20	120.563
C7—C15—C11	120.18 (18)	C20—C21—H21	120.849
N4—C16—C8	113.11 (17)	C22—C21—H21	120.854
N4—C17—C18	115.94 (17)	N5—C22—H22	118.151
N5—C18—C17	116.4 (2)	C21—C22—H22	118.151
N5—C18—C19	121.2 (2)	N4—C23—H23A	108.897
C17—C18—C19	122.4 (2)	N4—C23—H23B	108.899
C18—C19—C20	120.0 (2)	C24—C23—H23A	108.892
C19—C20—C21	118.9 (3)	C24—C23—H23B	108.896
C20—C21—C22	118.3 (3)	H23A—C23—H23B	107.725
N5—C22—C21	123.7 (2)	C24—C25—H25	120.400
N4—C23—C24	113.4 (2)	C26—C25—H25	120.399
N6—C24—C23	115.3 (2)	C25—C26—H26	120.492
N6—C24—C25	122.17 (18)	C27—C26—H26	120.490
C23—C24—C25	122.53 (19)	C26—C27—H27	121.022
C24—C25—C26	119.2 (2)	C28—C27—H27	121.032
C25—C26—C27	119.0 (3)	N6—C28—H28	117.816
C26—C27—C28	117.95 (19)	C27—C28—H28	117.809
			
C14—N3—C15—C7	−179.57 (17)	C8—C9—C10—C11	0.9 (3)
C14—N3—C15—C11	−0.6 (3)	Cl1—C10—C11—C12	−1.0 (2)
C15—N3—C14—C13	0.1 (3)	Cl1—C10—C11—C15	179.37 (11)
C16—N4—C17—C18	−70.7 (2)	C9—C10—C11—C12	178.57 (16)
C17—N4—C16—C8	−65.3 (2)	C9—C10—C11—C15	−1.1 (3)
C16—N4—C23—C24	−72.8 (2)	C10—C11—C12—C13	−179.91 (16)
C23—N4—C16—C8	166.49 (16)	C10—C11—C15—N3	−179.63 (15)
C17—N4—C23—C24	158.23 (17)	C10—C11—C15—C7	−0.7 (2)
C23—N4—C17—C18	57.1 (2)	C12—C11—C15—N3	0.7 (2)
C18—N5—C22—C21	−0.4 (2)	C12—C11—C15—C7	179.66 (15)
C22—N5—C18—C17	−178.93 (13)	C15—C11—C12—C13	−0.3 (3)
C22—N5—C18—C19	0.8 (2)	C11—C12—C13—C14	−0.2 (3)
C24—N6—C28—C27	1.1 (3)	C12—C13—C14—N3	0.4 (4)
C28—N6—C24—C23	178.99 (18)	N4—C17—C18—N5	75.0 (2)
C28—N6—C24—C25	1.3 (3)	N4—C17—C18—C19	−104.73 (19)
O2—C7—C8—C9	177.87 (14)	N5—C18—C19—C20	−0.3 (2)
O2—C7—C8—C16	−3.8 (3)	C17—C18—C19—C20	179.38 (13)
O2—C7—C15—N3	1.0 (2)	C18—C19—C20—C21	−0.5 (3)
O2—C7—C15—C11	−177.99 (13)	C19—C20—C21—C22	0.9 (3)
C8—C7—C15—N3	−178.35 (15)	C20—C21—C22—N5	−0.4 (3)
C8—C7—C15—C11	2.7 (2)	N4—C23—C24—N6	152.46 (19)
C15—C7—C8—C9	−2.8 (2)	N4—C23—C24—C25	−29.9 (3)
C15—C7—C8—C16	175.54 (14)	N6—C24—C25—C26	−2.4 (4)
C7—C8—C9—C10	1.1 (3)	C23—C24—C25—C26	−179.9 (2)
C7—C8—C16—N4	107.8 (2)	C24—C25—C26—C27	1.0 (4)
C9—C8—C16—N4	−73.8 (2)	C25—C26—C27—C28	1.2 (4)
C16—C8—C9—C10	−177.37 (15)	C26—C27—C28—N6	−2.3 (4)
C8—C9—C10—Cl1	−179.55 (14)		

### Hydrogen-bond geometry (Å, º)

Cg2 and Cg3 are the centroids of rings N5/C18–C22 and N6/C24–C28, respectively.

**Table:**

*D*—H···*A*	*D*—H	H···*A*	*D*···*A*	*D*—H···*A*
O2—H2···N5	1.04 (3)	1.66 (4)	2.689 (3)	168 (2)
C22—H22···O2^i^	0.93	2.46	3.348 (3)	160
C27—H27···N3^ii^	0.93	2.55	3.406 (3)	153
C17—H17b···*Cg*2^iii^	0.97	2.79	3.599 (3)	141
C23—H23*A*···*Cg*3^iv^	0.97	2.86	3.770 (3)	156

Symmetry codes: (i) −*x*+1, −*y*+1, −*z*; (ii) *x*, *y*+1, *z*+1; (iii) −*x*, −*y*+1, −*z*; (iv) −*x*, −*y*+2, −*z*+1.
